# Challenges of online learning amid the COVID-19: College students’ perspective

**DOI:** 10.3389/fpsyg.2022.1037311

**Published:** 2022-12-22

**Authors:** Yuefan Xia, Yawen Hu, Chenyi Wu, Ling Yang, Man Lei

**Affiliations:** ^1^Education College, Shanghai Normal University, Shanghai, China; ^2^Environmental and Geographical College, Shanghai Normal University, Shanghai, China; ^3^Foreign Languages College, Shanghai Normal University, Shanghai, China

**Keywords:** COVID-19, online learning, college students, challenges, students’ engagement, relationships, students’ feelings

## Abstract

Universities in China’s transition to online education in response to the COVID-19 pandemic have spawned several research studies. However, studies exploring college students’ technological skills, relationships with their peers and instructors, and collaborative learning experiences during the pandemic are scarce. Three aspects were explored in this mixed study: (1) changes in students’ engagement in class and the main factors involved; (2) students’ feelings and reactions during online learning; and (3) how students related to their peers and instructors. Data were collected through a qualitative survey supplemented by quantitative data about students’ attitudes to online learning using the SAROL scale. This paper argues that online learning may not produce the desired results due to lack of interaction with instructors, no campus socialization or well-trained technology skills, and appropriate content for online courses and group work. The findings further revealed that online learning offers college students new ways to learn independently, collaborate and build relationships with their peers. It encourages them to reconsider ways to improve their technology skills, learning methods, communication skills and reconceptualize their responsibilities as team members.

## 1. Introduction

The sudden outbreak of COVID-19 has affected the lives of people all over the world since 2019 (Ayittey et al., 2020; Villela et al., 2021). Health and safety concerns forced many schools to close temporarily (Jena, 2020). In China, the need for online learning increased rapidly, causing the traditional face-to-face learning mode to change to online learning as educators have strived to ensure students receive their formal education programs (Lei and Medwell, 2021).

The pandemic has brought unprecedented challenges to the education system, placing higher demands on emergency preparations as schools need to adapt to the changing environment and repeated outbreaks (Xue et al., 2020) - the so-called “new normal” (Wang, 2020 p.11). Educational institutions struggle to find alternative options to face-to-face education to deal with this challenging situation (Rieley, 2020). They shut down campuses to enable students keep a social distance from each other (Toquero, 2020). However, it is impossible to make a smooth transition from a traditional educational environment to online learning in a very short time. The rapid transition has brought many obstacles and challenges (Crawford et al., 2020). Students appear unable to understand the educational role of online technologies and consider them irrelevant or even an obstacle to learning (Ginns and Ellis, 2007; Ellis and Bliuc, 2019). For instance, on a learning platform called Xuexitong, the target students were not involved in virtual class activities and unable to achieve the desired improvement to their studies (Lei and Medwell, 2021). Cui et al. (2020) study showed that the proportion of students who completed their courses and homework on time decreased over time. Although the strength of the impact of the COVID-19 outbreak on education may take time to become fully apparent, educational institutions around the world are currently doing everything they can to create better online learning environments and resources for students in all academic fields by utilizing their limited resources to their utmost (Kaur, 2020).

An important aspect of assessing online learning is discovering how to identify problems from a student’s viewpoint in order to improve the quality of online courses. Students’ perspectives are invaluable, and their first-hand input comes from their experiences and expectations (Dawson et al., 2019). Furthermore, how college students reacted to online courses during the epidemic plays a crucial role in helping education professionals to meet the learning needs of students better in future when teaching modes of delivery change and new technologies emerge (Crews and Butterfield, 2014; Van Wart et al., 2020). Therefore, it is essential that the students’ perspective is central. Pragmatically, many valuable and practical findings and insights have been achieved through studies on teachers’ teaching efficiency, constraints, and challenges during COVID-19 (Arora and Chauhan, 2021; Ober et al., 2022). However, the students’ standpoint has received less attention than the teachers’ perspective in the assessment of online education’s effectiveness presented in previous studies.

The findings of this study can give university administrators and teachers a better understanding of what needs to be done to adjust to the future of online learning and help students overcome common challenges they are likely to face so they have a better learning experience. Due to the sudden transition in learning mode and learning environment, we consider in-depth insights into college students’ feelings and in-class performance, vital. Our study addresses the following research questions:

1. What factors affected students’ engagement in online learning?
2. What were college students’ feelings/reactions during online learning?
3. How did college students’ relationships with their peers and instructors change during online learning?

## 2. Literature review

### 2.1. Challenges presented by online learning before COVID-19

The rapid development of electronic technologies has made distance education easier (McBrien et al., 2009), but sometimes there can be many obstacles. Often, difficulties and problems associated with modern technology come from downloading errors, issues with installation, login problems, problems with audio and video, etc. Previous research has shown that some features like file sharing, whiteboards, and annotation are not easy to use, resulting in the underutilization of conferencing functions (Ming et al., 2021). In asynchronous learning environments, learning content cannot be provided in the same format as in offline classes, that is, it is impossible to provide real-time feedback and responses (Littlefield, 2018). At the same time, students feel a lack of learning community, experience technical problems, and have difficulty understanding instructional goals, which are the major barriers to online learning (Song et al., 2004). It is worth mentioning that certain challenges experienced in online courses are due to educators’ lack of online teaching skills or lesson preparation in the form of detailed teaching plans, lack of appropriate support from technical teams, and traffic overload in online education platforms.

One big problem of online courses is the monotonous learning scenario and the easy visual fatigue of the learners (Zhou and Ren, 2019). Sometimes, students found online teaching boring and unappealing because online teaching videos were too long, reducing learners’ enthusiasm and interests in learning (Li and Wang, 2019). Although asynchronous online learning provides a lot of response time and high degrees of flexibility for students, they still have difficulty finding enough time to complete tasks (Knox, 2016). Moreover, mediocre course content is also a major issue. Students’ level of preparedness in using Learning Management Systems (Parkes et al., 2014) is low. Online programs need to be designed to be creative, interactive, relevant, student-centered, and group-based (Partlow and Gibbs, 2003).

Not only teachers but students also face challenges due to a lack of appropriate learning materials, their attitude to learning, lack of self-discipline, and the inadequate learning environment in some of their homes during self-isolation (Brazendale et al., 2017). Furthermore, Willging and Johnson (2009) found that students may not have been interested in the learning materials used because they lacked pre-knowledge of the course, so were unable to follow the learning material offered (Pierrakeas et al., 2004).

### 2.2. Challenges of online learning during COVID-19 isolation

COVID-19 was a blow to traditional learning methods in academic institutions around the world. The administration systems of educational institutions around the world chose online course tuition to restore education provision when the physical presence of students and tutors was impossible. Online learning during COVID-19 could be delivered synchronously or asynchronously. Obviously, the current situation is not like traditional online learning but more like crisis learning and has posed huge challenges for students. They may be faced with unstable Internet connections, which makes it impossible to ensure equity between students through online learning (la Velle et al., 2020; Xue et al., 2020). At the same time, this causes attendance and engagement issues in online sessions, so online education can be less adaptable than supposed. Moreover, students had to rapidly turn to unfamiliar learning methods, while responding as individuals and members of social groups to the impact of the epidemic on their daily lives, physical and mental health (Macintyre et al., 2020). It is not hard to understand why teachers’ techno-pedagogical skills appear to be the major factor affecting student engagement during this time. Researchers have found a positive correlation between the students’ grades and their technological abilities—if teachers are not proficient in using the functions of network equipment, students’ learning is correspondingly negatively affected (Masry-Herzallh and Stavissky, 2021). Therefore, in future, teachers need to improve their teaching skills to facilitate the transfer of knowledge and their communication with students (Palanisamy et al., 2020), and it is necessary to explore online teaching strategies that focus on students’ interests as a way to ensure higher levels of student engagement.

Most importantly, the uncertainty about when the outbreak restrictions will end has led to much anxiety and fear among students isolated at home. Research has revealed that personal challenges (such as economic and psychological stress) have reduced students’ willingness to learn online in future, while the quality of the online experience (including instructional and assessment quality) has improved their attitude to learning online in future (Al-Salman and Haider, 2021). Therefore, teachers need to communicate with their students regularly to help alleviate any inner turmoil and cater to their other psychological needs during these stressful times (Anderson, 2020; Snelling and Fingal, 2020; Tate, 2020). It is suggested that closely monitoring students’ feelings can have a positive impact on their learning (Morgan, 2020).

### 2.3. Effectiveness of online learning

While online learning has been shown to help protect students and faculty from infection during the COVID-19 pandemic, it has not been as effective as traditional learning. Five common criteria have been proposed for assessing the effectiveness of the digital transformation in higher education institutions; the changes, their speed, technology involved, users and system capacity, and economic implications (Kopp et al., 2019). Online learning means the use of technological devices, the Internet as a tool. Adedoyin and Soykan’s research (Adedoyin and Soykan, 2020) noted that technical issues, socio-economic factors, human and pet intrusion, digital competence, assessment and supervision, and heavy workload can affect the effectiveness of online learning.

The intervention of teachers can improve students’ learning efficiency to a certain extent. Ahmad’s (2020) study found that most students struggled with online learning, particularly in underdeveloped locations with poor connectivity (Ming et al., 2021). In addition, the content of the online course material discussed in class requires students to type messages through the chat box of the virtual conferencing applications, which requires responding within time limits (Zhong, 2020).

### 2.4. Changes of students’ relationships with others in online learning

Online learning lacks the physical presence of a face-to-face interactive relationship between fellow students, and students and their educators (Means et al., 2009; Alawamleh et al., 2020), so how students and instructors interact and how students collaborate with each other has to change. Although there are a variety of online applications, many tutors cannot provide students with remote care and timely feedback on their academic performance (Collazos et al., 2021). This makes students dissatisfied with online learning. Research has found that Arab students, for instance, have negative feelings about online learning (Masry-Herzallh and Stavissky, 2021). Likewise, college students from Pakistan perceive conventional learning as more motivating than online learning; for example, they enjoy participating in conventional learning activities and become more easily immersed in the atmosphere of conventional interaction (Muhammad and Kainat, 2020). In essence, students are “social learners” who long for interaction with their peers and instructors; they can be easily distracted and pay less attention to the content of online courses (Bozkurt and Sharma, 2020) and have difficulty maintaining self-discipline (Nishimwe et al., 2022). Generally, students tend to prefer face-to-face teaching and learning.

Specifically, research has found that the learning performance of students who participated in online discussion activities was significantly better than those who did not, even when their other learning experiences were similar (Du et al., 2019). Collaborative learning among peers can also facilitate the exchange of ideas and information to improve their knowledge level (Luaran et al., 2014). However, group collaborative learning appears to be less effective because students have weak cooperative aims (Stahl, 2005; Collazos et al., 2007). Therefore, many instructors employed more technology and applications for synchronous learning to increase student motivation and improve learning efficiency and learning achievement (e.g., PowerPoint voiceover slides for uploading course content, using the lounge feature in video conferencing to increase interaction with students and encourage communication between students, using WeChat for class discussion and group collaboration; Gao and Zhang, 2020; Farrell and Stanclik, 2021; Gan et al., 2022; Moorhouse and Wong, 2022).

## 3. Methodology

The purpose of the study is to explore the effectiveness and challenges of online learning during the COVID-19 pandemic and to propose possible solutions derived from analyzing the underlying causes of the challenges faced by higher education students in an eastern city in China. The present study employed a mixed-mode research design to answer the research questions. The researchers used a qualitative survey as the primary data collection tool and supplemented it with quantitative data from a students’ attitudes regarding online learning (SAROL) scale (Muhammad and Kainat, 2020) to investigate the attitudes of Chinese higher education students during the COVID-19 pandemic to the online learning mode compared to the traditional learning mode, as well as the challenges and opportunities this new online learning mode presented.

### 3.1. Sampling strategy and participants

The researchers used purposive sampling for the qualitative phase by sending an invitation email to college students who were following online courses using online learning platforms like Tencent Conference and Xuexitong. There were 102 male and 128 female participants from five universities in an eastern city in China. The participants’ ages ranged from 18 to 20 years old. Snowball sampling was also adopted in the quantitative phase. The researchers shared the questionnaire links with currently enrolled research participants and encouraged them to spread the project on social media platforms such as WeChat, QQ, and Weibo to capture a growing chain of participants (Creswell, 2011).

### 3.2. Data collection instruments

Data were collected through a demographic questionnaire, qualitative survey, and a SAROL instrument. The demographic section included questions about the participants’ age, gender, grades, and online learning experience.

The qualitative survey adopted a semi-structured interview (Bryman, 2016) that focused on students’ course engagement, relationship with their peers and instructors, their experience of collaborative learning, and the effectiveness of online learning. Research instruments were designed collaboratively by the researchers. During the pilot, researchers wrote open questions about students’ online engagement, students’ relatedness, and the experience of collaborative learning from the perception of students. For example, “what do you think has affected your group work completion?” The research instruments were then refined into several main themes—views on learning, collaborative learning, and active learning. These are detailed in Table 1.

**Table 1:** Interview questions.

Questions	Additional follow-up
1. What kind of online activities have been included in your module/s?	Have you had any virtual conferencing applications? Or learn without paper? Or other online learning resources?
2. How would you describe the difference between online learning and offline learning?	
3. What are the benefits and reasons for students to engage with online learning activities?	
4. What kind of learning makes you more motivated to learn well?	
5. What are the reasons that you don’t engage with the online activities?	Think about how the following might or might not impact student engagement in online activities:
	• The shyness to turn on the video
	• How prepared students are to learn independently
	• How involved the tutor is in the online activity
6. What are reasons that impact your collaborative activities or completion of group works?	
7. What are the most effective and minimal aspects in online activities?	

To explore the effectiveness and challenges of online learning from the perception of college students, we modified the SAROL scale from Muhammad and Kainat’s (2020) study to investigate students’ attitudes toward online learning during the epidemic. SAROL has been widely used to explore higher education students’ responses to online learning (e.g., Coman et al., 2020; Guo et al., 2020; Serhan, 2020). The questionnaire consists of eight questions, the first question multiple-choice to elicit the major challenges of online education during the COVID-19 outbreak, the second to eighth rated on the Likert Scale as strongly agreeing, generally agreeing, or disagreeing to investigate the respondents’ attitudes toward online teaching. The questionnaire format and items were piloted and revised with a group of 20 students at S University (anonymized). The researchers used the SAROL results to better understand participants’ responses to the open-ended questions.

#### 3.2.1. Modifications to the SAROL scale

Considering the differences in cultural background and technological level between Pakistan and China, the necessary modifications were made.

Online learning can be effective in digitally developed countries, such as China. However, in some underdeveloped countries, such as Pakistan, much of the learning and teaching, as well as the management of academic institutions, is handled manually. The lack of fast, affordable, and reliable Internet connections has hampered the progress of online learning in that context. The original questionnaire cited by Muhammad and Kainat (2020) asked students in Pakistan about their attitudes toward online learning based on factors about students’ limited access to the Internet, such as inability to use electronic devices and price; these factors were removed in the context of this research based in China. Familiarity with online functions, privacy concerns, and signal strength issues were added to illustrate the main reasons for the low frequency of online learning software functions.

While the COVID-19 pandemic has prompted Chinese universities to turn to online education, little is known about the impact of students’ skill in using virtual conferencing functions on their views of teaching quality. Therefore, the researchers changed the second question to “I am proficient with conferencing applications functions.” Table 2 shows the questionnaire’s final version.

**Table 2:** Students’ attitudes regarding online learning.

Attitudes	No. (%)
1. The main reasons for the low use frequency of conferencing applications functions	
Not familiar with the function	12 (5.22)
Shy/afraid of exposing your privacy	160 (69.57)
Signal reception/strength problem	48 (20.87)
For fear of exposing their privacy	10 (4.35)
2. I am proficient with conferencing applications functions	
Agree	94 (40.87)
Somewhat agree	115 (50)
Disagree	21 (9.13)
3. I am comfortable communicating electronically	
Agree	105 (45.65)
Somewhat agree	108 (46.96)
Disagree	17 (7.39)
4. No difference between online and conventional learning	
Agree	28 (12.17)
Somewhat agree	67 (29.13)
Disagree	135 (58.7)
5. Online learning is more motivating than conventional learning	
Agree	43 (18.7)
Somewhat agree	71 (30.87)
Disagree	116 (50.43)
6. Complete university course can be completed effectively through internet	
Agree	51 (22.17)
Somewhat agree	76 (33.04)
Disagree	103 (44.78)
7. It is easy to complete group projects /assignments digitally	
Agree	54 (23.48)
Somewhat agree	132 (57.39)
Disagree	44 (19.13)
8. Face-to-face contact with the instructor is necessary for learning	
Agree	158 (68.70)
Somewhat agree	58 (25.22)
Disagree	14 (6.09)

To exclude the interference of gender in this study, the researchers conducted a Chi-square test. The results in Table 3 indicate no significant difference between gender and students’ attitudes to online learning [*r* (230) = 0.93, *p* > 0.05, representing a small effect].

**Table 3:** Pearson correlation; Sarol and gender.

	Value	*df*	Progressive Sig. (2-tailed)	Precise Sig. (2-tailed)	Precise Sig. (1-tailed)	Point probability
Pearson correlation	0.937¹	3	0.816	0.833		
Likelihood ratio	0.962	3	0.810	0.821		
Fisher	1.157			0.807		
Linear and linear combinations	0.737³	1	0.391^2^	0.412	0.248	0.097
McNemar–Bowker						
*N*	230					

^1^Cell 2 (25%) has an expected count of less than 5. The minimum expected count is 1.17.

^2^Calculated for *p* × *p* tables only (*p* > 1).

^3^Standardized statistics are 0.859.

#### 3.2.2. Refining the scale

An item analysis was done to remove any items which did not meet the statistical standard. Of the 7 items in the initial SAROL scale, items VarA2 and VarA8’s association with the total score of all variables were 0.269 and − 0.166, so each failed to reach the required level of significance (*p* < 0.01) and were removed. The remaining 5 items demonstrated good differentiation.

**Figure 1:**
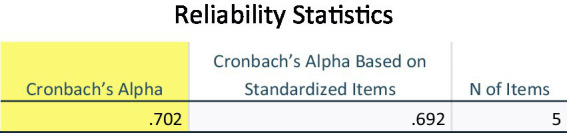
Cronbach’s alpha.

In this study, the reliability analysis was done according to the SAROL scale. The overall Cronbach’s α coefficient was 0.702, an acceptable internal reliability (Creswell, 2003), as shown in Figure 1, the internal consistency being ideal. After removing VarA2 and VarA8, total correlations of individual items were all higher than 0.4, while deleting the two items did not lead to an increase of Cronbach’s α coefficient. This indicated that the scale’s internal consistency and reliability were acceptable.

### 3.3. Data collection procedures

Questionnaires are considered a cost-effective and human resource-efficient method of data collection (Creswell, 2003). The researchers emailed potential participants information about the study and the link to the online questionnaire platform used to collect data. Consent forms were attached to the questionnaire stating that the information provided would only be used for academic and research purposes and assuring the respondents of their rights to privacy, to be informed, and of the confidentiality of the research (Creswell, 2003). The participants were informed their participation was voluntary, with the right to withdraw from the study at any stage. The second part of survey included demographic information, while the third and the final part consisted of the SAROL scale and the qualitative survey questions, respectively. The content of the interviews was recorded and transcribed professionally verbatim. Each participant was given a code to protect their identity (e.g., S1 stands for College Student 1) and participants were asked not to identify themselves to the others during recording. Sixteen participants agreed to participate in the survey and allowed their responses to be recorded.

### 3.4. Data analysis

#### 3.4.1. Qualitative data analysis

We used NVivo 12.0 to conduct a thematic data induction analysis of the recorded content (Braun and Clarke, 2006). We developed codes based on our literature review and research questions and modified codes when conducting the data analysis. We double coded the qualitative data to avoid unnecessary or duplicate codes, then organized the final codes into a thematic structure, and finally recoded the transcripts to ensure consistency. Examples are included in Table 4. This inductive approach was more appropriate to the contextual and exploratory nature of this research (Bryman, 2016).

**Table 4:** Recommendations for practice.

Active learning	Types of activity
	Level of challenge
	Task design
	Learning tool
	Online learning resources
Disengagement	Use of technology
	Stress and pressure ‘boring’
	Online course quality
Collaborative learning	Relatedness
Views of learning	Learning environment
	Learning socially
	Learning modes and styles
	Motivation

#### 3.4.2. Quantitative data analysis

The analysis of the reliability and validity of the data was completed using the Statistical Package of Social Science (SPSS 28.0) and all figures presented through excel. The percentage of students’ participation in collaborative activities and online learning and the frequency of using the conferencing applications functions were calculated through descriptive statistical analysis. Inferential analysis was used to assess the availability and convenience of online classes and the differences from traditional teaching, as the opportunities and challenges students experienced during the COVID-19 pandemic. To enhance the reliability and validity of this study, the data analysis was conducted individually by each of the researchers, followed by discussions to reach consensus on the results.

## 4. Results

The following sections show the results of the questionnaire and interviews given together to the participants who had been experiencing COVID-19 pandemic restrictions on physical content. The data and interview feedback revealed the obstacles to online learning and emotional feedback concerning online learning. Although the responses varied, three main themes emerged. We selected a representative sample of interviewees for each main theme to give an indication of the feelings surrounding them.

### 4.1. The challenge from poor student engagement in online learning

#### 4.1.1. Lack of technical skills

**Figure 2:**
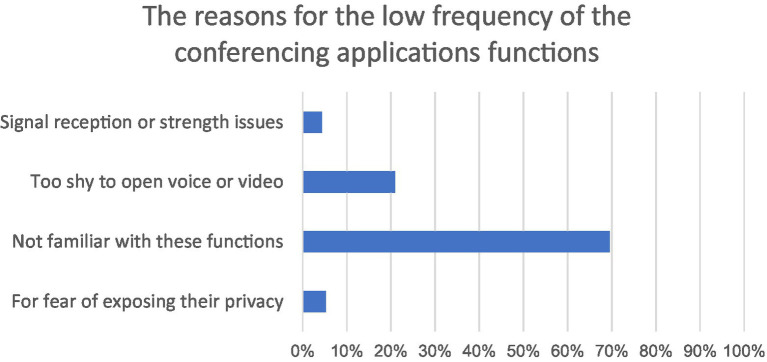
Distribution of reasons for the low frequency of the virtual conferencing tools.

**Figure 3:**
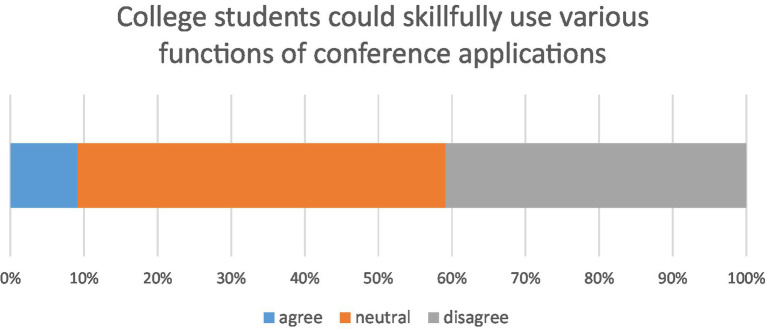
Distribution of user’s familiarity with the features on virtual conferencing tools.

The reasons for the low frequency of online participation also suggest why participants were reluctant to use conferencing functions during COVID-19. The reasons include unfamiliarity with online systems and lack of confidence, poor signal or strength issues, and fear of privacy exposure. Based on the results of the questionnaire, the main reason participants rarely used voice and screen sharing in conferencing applications was they felt too shy to speak (69.57%). The other primary reason for using functions less frequently was signal reception or strength issues. 20.87% of participants responded that they sometimes could not hear others’ voices, could not see others’ shared files, or videos would stall. Another problem was that participants were afraid their privacy might be invaded (4.35%) and they were unfamiliar with numerous functions of conferencing applications (5.22%). In other words, due to the sudden outbreak of the COVID-19 epidemic, students had no time to fully explore these features and they seldom used virtual conferencing applications in offline learning settings, always arranging a time to meet and discuss assignments in person. The findings strongly suggest participants preferred offline communication to online communication when they had to attend to online courses concerns due to online uncertainties. Figure 2 highlights the reasons for the low use of the current virtual conferencing applications (Tencent Meeting). Figure 3 is a bar chart illustrating user’s familiarity with the features on virtual conferencing tools. For example, S2 stated that:

We have some virtual conferencing applications like Tencent Conference. It is not completely paperless learning although I often learn with my computer and tablet, completing homework such as courseware on them. These technological devices bring convenience, but also require self-discipline and proper use, which can affect my participation and engagement in class. (S2).

#### 4.1.2. Low learning motivation

**Figure 4:**
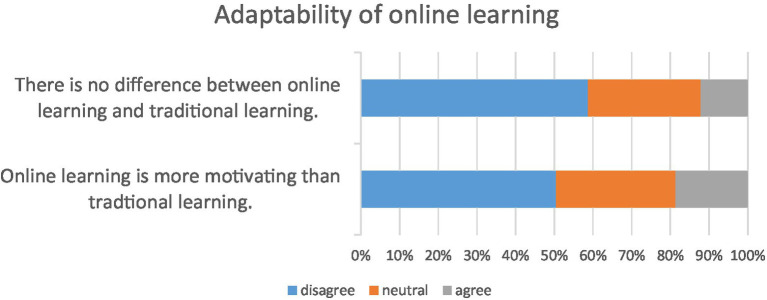
Bar chart of adaptability of online learning.

In response to the question of whether online and conventional learning are the same, 12.17% reported that online learning is very different from the conventional learning mode, while 58.7% felt that there was little difference between online and conventional learning. According to the questionnaire, only 18.7% of students felt that online learning was more motivating than conventional learning, while more than half the students (50.43%) disagreed that online learning was more motivating than conventional learning disrupted by the COVID-19 epidemic. Figure 4 shows the results of students’ motivation to learn during the COVID-19 pandemic.

### 4.2. What were college students’ feelings/reactions during online learning?

#### 4.2.1. Students’ feelings/reactions during online learning

**Figure 5:**
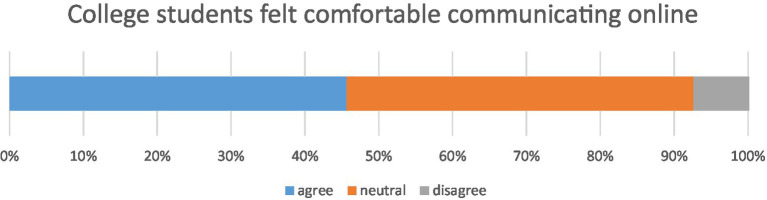
Distributions of whether user feel comfortable without voice or video during a virtual conference meeting.

The usability study asked participants whether they felt comfortable without voice or video features while performing collaborative activities or attending online courses. Almost half the responses (46.96%) were neutral, meaning for them, it did not matter if voice or video were on or off, while nearly half (45.65%) reported feeling more comfortable and relaxed (less nervous) without opening voice or video. Around 7.9% of participants experienced negative feelings, such as loneliness or boredom. To sum up, silent online communication seemed to alleviate anxiety during the COVID-19 pandemic, so participants preferred typed communication to online communication through voice and video, particularly during collaborative activities (when some group members did not know others very well) or attending subject classes. Figure 5 shows whether participants felt more relaxed by not opening voice or video during virtual conferencing. For example, referring to his/her feelings during online collaborative learning, as S5 stated that:

The access to and sharing of information and materials is more convenient, and I also communicate more closely with my team members. (S5).

#### 4.2.2. How did students value online learning?

The questionnaire asked participants about their experiences with online collaborative learning during the COVID-19 pandemic. 44.78% of respondents thought that it was very challenging to effectively complete entire college courses through online learning. Moreover, 57.39% of students reported that they felt difficult while doing group projects or assignments through distance learning, while 23.48% of students valued their online learning experience as they found conducting group projects or assignments digitally was easy in actual practice. As further illustrated in interviews, students acknowledged that their online learning experiences had “forced” them to “continually develop technological skills to function effortlessly” (S8) and “increased their awareness of participation in a digital world” (S13). Overall, the participants rated the collaborative experience to be neutral and the efficiency of collaborative learning and the mastery of course content could be challenging more than rewarding.

**Figure 6:**
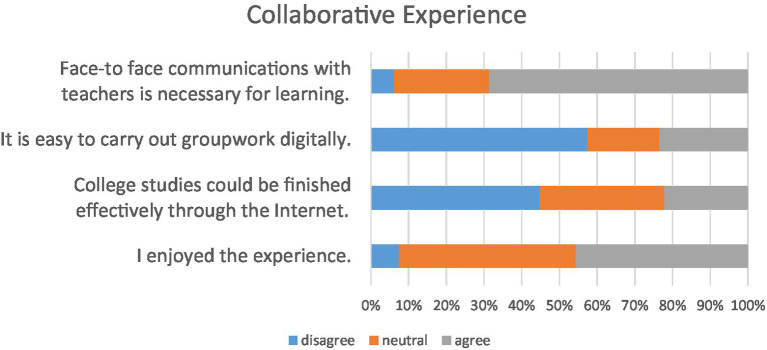
Distribution of students’ collaborative experience during online learning.

Figure 6 shows the distribution of collaborative experience gained through the uses of online course platforms. When making a reference to the effectiveness of face-to-face communications with teachers, 68.7% of students agreed that face-to-face communication with teachers is necessary for online learning, this emphasizes the importance of teacher’s presence. This sense of social presence could counteract students’ loneliness when a direct interpersonal touch is missing, which can be “extremely important during the current pandemic crisis” (S6).

### 4.3. How did college students’ relationships with their peers and instructors change during online learning?

#### 4.3.1. Face-to-face communications between college students and their instructors

Figure 6 shows the distribution of collaborative experiences gained during the use of online class platforms. While referring to the effectiveness of face-to-face communication with their instructors, 68.7% of the students felt that face-to-face contact with their instructors is necessary in order to learn. In traditional offline classes, college students could have face-to-face communication with their instructors and ask them for guidance, but on transferring to online learning, they could only communicate through social apps and online meetings. Communicating in real time with instructors seems to help college students to “better understand their instructors’ tasks and guiding concepts” (e.g., S7, S9, and S10):

I couldn’t finish some of my academic tasks without face-to-face communications with my instructor. Sometimes I cannot get a reply in good time from my instructors when asking online. (S7)

#### 4.3.2. Collaborative learning between peers

The majority of respondents considered that online learning is different from offline learning in terms of group collaboration. In the past, college students could conduct collaborative learning by booking group discussion rooms to complete groupwork, but after switching to online learning, they carry out collaborative learning in virtual meetings.

The questionnaire investigated the participants’ collaborative experience of online learning due to the impact of COVID-19 pandemic. 57.39% of students reported having difficulties doing group projects or assignments through online learning, while 23.48% felt able to easily finish group projects or assignments digitally. Overall, the participants rated the collaborative experience to be neutral, but the effectiveness of collaborative learning and the mastery of course content were problematic. S11’s quote is a typical example:

I think communication ability, team member responsibility, reasonable work distribution, management ability are the keys to collaborative learning. Without good communication skills, it is easy to cause internal strife, and team member’s poor sense of responsibility leads to low work involvement, which then affects the quality of work. (S11)

## 5. Discussion

The majority of the college students surveyed were not satisfied with online learning. Low engagement in online learning and the effectiveness of online learning were the major challenges faced by college students in this study. According to Means et al. (2009) and Alawamleh et al. (2020), the efficiency of online learning is questionable and there are many challenges to the success of online learning (Adedoyin and Soykan, 2020). This research also revealed an additional challenge faced by students, i.e., their relationships with instructors and peers.

Based on this research, lack of technology skills and low learning motivation result in students’ low engagement in online learning. Being shy or reticent about turning on voice and video is the main barrier faced by higher education students (69.57%) of S University, while unfamiliarity with virtual conferencing application functions, signal reception, strength issues, and fear of loss of privacy are additional obstacles; hence, full advantage of features in the virtual conferencing application is not taken. Students in Song et al.’s (2004) research encountered some additional technical problems like downloading errors, issues with installation, login problems with audio and video, etc. Ming et al. (2021) found that some features like file sharing, whiteboard, and annotation are not easy to use, resulting in the underapplication of conferencing functions. It is worth making clear that teachers’ mastery of technology also affects students’ engagement (Masry-Herzallh and Stavissky, 2021). Therefore, students need to overcome their shyness in front of the camera, while teachers need to explore and expand their online learning strategies.

One of the less discussed areas of online education is the need to motivate students to learn online. 50.43% of participants indicated they felt a strong incentive for to learn offline. This concurs with Muhammad and Kainat’s (2020) conclusion that conventional learning was more motivating than online learning. In traditional classes, students more easily immersed themselves and participated in academic tasks actively through their face-to-face engagement with teachers. Furthermore, students believed that they cannot do their homework effectively and on time without checks and mandatory provisions by teachers, hence their tendency to procrastinate.

This research indicated that conventional learning was more effective than online learning, the same as Kopp et al.’s (2019) findings. While comparing the effectiveness of conventional and online learning, 68.7% of respondents felt that face-to-face communication with their teachers was crucial to effective learning. According to our questionnaire, 44.78% of students reported being unable to complete entire college online courses effectively through online learning. Also, most of the interviewees surveyed preferred offline learning. They asserted the most effective aspect of online learning was the easily accessible learning resources, and the least productive aspect is the lack of supervision. Such reactions have been explained by distraction (Bozkurt and Sharma, 2020) and lack of discipline (Nishimwe et al., 2022).

The majority of participants reflected meeting great challenges in the process of online learning. Only by switching off voice and video, did the surveyed students (45.65%) feel comfortable. A minority (7.9%) experienced negative feelings, such as loneliness or boredom, which made them sometimes uncomfortable and reduced their passion for online learning. This result is explained by the fact that today’s students seem to be shy and prefer to be alone, so shutting down video and voice functions makes them feel safer and more relaxed. However, if lack of face-to-face social interaction continues, students may suffer psychological distress at all levels (McCarthy, 2020). According to Macintyre et al. (2020), the epidemic impacts students’ daily lives, and their physical and mental health.

What’ s more, lack of face-to-face communication and collaborative learning with peers and instructors is an extra barrier, challenging college students’ relationships with their instructors and peers, even though group work online can be as effective as face-to-face learning (Ocker and Yaverbaum, 1999). Due to physical limitations caused by the pandemic, 57.39% of the students think that they have difficulty in completing group projects because group study is boring and unappealing. Group study online needs to be (re)designed to be creative, interactive, relevant, student-centered, and group-based, as suggested by Partlow and Gibbs (2003). Lack of appropriate support from instructors makes work more time-consuming, thus the importance of clear and relevant instructions in group study cannot be ignored. However, previous studies (Gao and Zhang, 2020; Farrell and Stanclik, 2021) have shown how several instructors have made the most of technology (e.g., PowerPoint voiceover slides WeChat, and so on) to address the challenge of online communication and instruction.

## 6. Conclusion

Although online learning can help safeguard the health of students and faculty, it has proven to be less successful than traditional learning. The amount of student-teacher contact and campus socialization, level of technical competence, and appropriateness of learning content for online courses and group work are key factors for whether or not online learning produces the desired results. Therefore, students’ poor performance in online learning can be partly due to their dissatisfaction with the format and quality of course delivery and lack of interaction with others, leading to boredom and low motivation to learn. The findings of our study have revealed that online learning offers college students a new way to learn independently and to collaborate and build relationships with peers, which can encourage students to reconsider how to improve their technical skills, learning methods, and communication skills and review their responsibilities as team members. Technical skills training in future should be given to both faculty and students in order to improve students’ proficiency in applied skills and eliminate communication barriers based on poor skills. It is also advisable to allow students more time to find online learning methods that work for them and to provide them with guidance for following learning materials in to improve their learning efficiency and understanding and application of the content. What is more, teachers should improve their pedagogical skills and applications to increase the frequency of interaction with students by regularly checking their production and providing feedback on students’ academic performance as well as responding to psychological problems. Similarly, in a collaborative learning environment, students themselves need to develop more techniques to improve their communication with other class members and, most importantly, they need to develop a positive attitude toward group work, increase their own sense of team responsibility, and actively participate in group discussions and task completion during this difficult time. The authors hope that these findings will help students who need to learn online to better address similar challenges they encounter, since some new forms of learning, such as “blended learning” and “project-based learning,” are likely to continue to exist in post-epidemic learning.

The study’s greatest limitation is that it addresses the situation in one eastern city in China, so it is impossible to make broad claims. In the event of the epidemic’s resurgence in China, the researchers have had no opportunity to interview more college students, meaning the existing questionnaire data may not as comprehensive and detailed as desirable. To obtain broader and more reliable results, the design of the questionnaire could be improved, and more comparative studies could be conducted with colleges students in other contexts to better understand the similarities and differences through a larger capacity in sample files.

However, even though the sample size is small, the results can shed light on common challenges that students experienced in online class during COVID-19 pandemic. Understanding how students and their instructors perceive the online mode of higher education instruction in China can aid the development of more efficient ways of taking online classes and adapting better to online learning. There was a lot of agreement between students and instructors when it came to their impressions of online learning. The students and teachers’ views reflected and bolstered each other’s, so this level of agreement provides a basis for designing new online courses and improving the online teaching and learning experience.

## Data availability statement

The original contributions presented in the study are included in the article/supplementary material, further inquiries can be directed to the corresponding author.

## Ethics statement

The studies involving human participants were reviewed and approved by Shanghai Normal University. The patients/participants provided their written informed consent to participate in this study.

## Author contributions

YX, YH, CW, and LY are undergraduates, and ML is an Assistant Professor. ML has made substantial contributions to the conception and design of the work. She supervised the project and designed the theoretical framework, and research methods of the manuscript. She has contributed to the revision of the manuscript, to the acquisition, analysis, and interpretation of data for the work. YX has made great contributions to the design of the research framework and has organized the database, drafted, and written the abstract, literature review, introduction, and conclusion. YH has written the discussion section. CW has drafted and written the interpretation of data of this manuscript. LY has helped to perform the statistical analysis and written the methodology. All authors have collected the data, helped write the first draft of the manuscript, revised the manuscript several times and approved the submitted version.

## Funding

This research was sponsored by the research project “Exploring the reform and latest practice of teacher education” which was sponsored by Foreign Languages College, Shanghai Normal University.

## Conflict of interest

The authors declare that the research was conducted in the absence of any commercial or financial relationships that could be construed as a potential conflict of interest.

## Publisher’s note

All claims expressed in this article are solely those of the authors and do not necessarily represent those of their affiliated organizations, or those of the publisher, the editors and the reviewers. Any product that may be evaluated in this article, or claim that may be made by its manufacturer, is not guaranteed or endorsed by the publisher.
